# Notch信号通路在小细胞肺癌中作用的研究进展

**DOI:** 10.3779/j.issn.1009-3419.2025.106.17

**Published:** 2025-07-20

**Authors:** Feixue GU, Kaiyue ZHAO, Hengshuo YAN, Dongxin SUI

**Affiliations:** ^1^250033 济南，山东大学第二临床学院; ^1^The Second Clinical College, Shandong University, Jinan 250033, China; ^2^250033 济南，山东大学第二医院呼吸内科; ^2^Department of Respiratory Medicine, Second Hospital of Shandong University, Jinan 250033, China

**Keywords:** 肺肿瘤, Notch信号通路, 分子机制, Lung neoplasms, Notch signaling pathway, Molecular mechanisms

## Abstract

小细胞肺癌（small cell lung cancer, SCLC）是一种起源于肺神经内分泌细胞的高度侵袭性肺癌亚型，占肺癌总数的10%-15%，其特点是早期转移率高和预后极差，并常伴随耐药性和复发的挑战。研究表明，Notch信号通路通过调控细胞增殖、分化和凋亡等过程，在SCLC的发生和发展中发挥着关键作用。在SCLC中Notch信号的异常可能促进肿瘤恶性转化及耐药性的发生。此外，Notch通路还参与SCLC的上皮-间质转化，并通过与肿瘤免疫微环境的相互作用影响免疫逃逸机制。本文综述了Notch信号在SCLC中的分子机制，包括其受体与配体的作用、信号转导过程及在肿瘤发生中的角色，同时讨论了其作为潜在治疗靶点的研究进展，并展望该领域未来的研究方向。

## Abstract (en)

Small cell lung cancer (SCLC), a highly aggressive subtype of lung cancer that originates from pulmonary neuroendocrine cells, accounts for 10% to 15% of all lung cancers. It is characterized by a high rate of early metastasis and extremely poor prognosis, often accompanied by challenges such as drug resistance and recurrence. Related researches indicates that the Notch signaling pathway plays a crucial role in the occurrence and development of SCLC by regulating processes such as cell proliferation, differentiation, and apoptosis. In SCLC, abnormal Notch signaling may promote tumor malignancy and the occurrence of drug resistance. Additionally, the Notch pathway is involved in the epithelial-mesenchymal transition of SCLC and influences immune escape mechanisms through interactions with the tumor immune microenvironment. This article reviews the molecular mechanisms of Notch signaling in SCLC, including the roles of its receptors and ligands, signal transduction processes, and its role in tumorigenesis. It also discusses the research progress of Notch signaling as a potential therapeutic target and looks forward to future research directions in this field.

小细胞肺癌（small cell lung cancer, SCLC）作为一种高度侵袭性的肺癌，以快速生长和早期转移为特征^[1]^。Notch信号通路在SCLC的发生和发展中发挥着重要作用。在诊断时，近2/3的SCLC患者已处于广泛期，中位总生存期少于12个月^[2]^。铂类药物与拓扑异构酶抑制剂联合治疗是当前SCLC化疗的首选方案。放疗能提高局限期SCLC患者的局部控制率，但对广泛期SCLC患者的生存获益有限^[3]^。尽管SCLC对放疗和化疗高度敏感，但患者在初始治疗后复发率较高，导致治疗效果有限^[4]^。

鉴于现有治疗方法面临耐药性、治疗副作用等挑战，亟需开发新的靶向治疗策略。Notch信号通路的失调与SCLC的发生与发展密切相关。研究^[5]^表明，Notch信号通路是SCLC患者接受免疫检查点抑制剂（immune checkpoint inhibitors, ICIs）治疗临床获益最显著的预测因子。靶向Notch通路的药物已在多种肿瘤治疗中展现出良好的应用前景。因此，深入研究Notch通路在SCLC中的具体机制，不仅有助于揭示该通路在SCLC中的重要作用，还可能为其治疗提供全新的靶点，从而为临床提供更精准、有效的治疗策略。本文旨在综述Notch信号通路在SCLC中的作用机制，并讨论其作为潜在治疗靶点的研究进展，以期为SCLC治疗提供新的思路。

## 1 Notch信号通路概述

Notch信号通路由四种受体（Notch1、Notch2、Notch3、Notch4）、五种配体[Jagged家族（JAG1、JAG2）和Delta家族（DLL1、DLL3、DLL4）]及其下游效应分子组成。Notch受体为跨膜蛋白，主要由细胞外结构域（Notch extracellular domain, NECD）、跨膜结构域（transmembrane domain, TMD）和细胞内结构域（Notch intracellular domain, NICD）三部分组成。NECD包含29-36个表皮生长因子样重复序列和1个负调控区，这些结构通过O-糖基化修饰调节受体对不同配体的亲和力^[6]^。

Notch受体合成后在高尔基体中被Furin蛋白酶切割S1位点。切割后的受体与配体结合后，其S2切割位点被去整合素-金属蛋白酶（a disintegrin and metallo-proteinases, ADAM）家族成员（特别是ADAM10或ADAM17）切割，释放细胞外片段，随后其S3切割位点被γ-分泌酶复合体切割，促使Notch受体可溶性NICD释放。当NICD被切割并转运至细胞核后，在转录共激活因子（mastermind like transcriptional coactivator, MAML）等的参与下，NICD的RAM结构域与CSL结合，促使CSL转变为转录激活因子，从而激活发状分裂相关增强子（hairy and enhancer of split, HES）、碱性螺旋-环-螺旋转录因子基因（hairy and enhancer of split related with YRPW motif, HEY）等靶基因^[7]^。具体作用机制见图1。

**图1:**
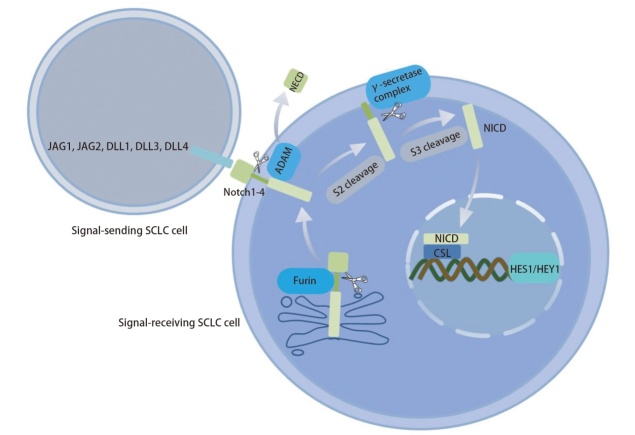
Notch信号通路激活过程。Notch受体合成后在高尔基体中被Furin蛋白酶切割S1位点。受体与配体结合后，该受体的S2位点被ADAM切割，释放胞外片段；随后γ-分泌酶复合体切割其S3位点，释放可溶性NICD。NICD转运入核后与CSL结合，从而激活HES、HEY等靶基因。

激活发状分裂相关增强子1（hairy and enhancer of split 1, HES1）和碱性螺旋-环-螺旋转录因子基因1（hairy and enhancer of split related with YRPW motif 1, HEY1）的激活会抑制ASCL1，ASCL1是SCLC神经内分泌（neuroendocrine, NE）分化的关键调节因子，在SCLC中通过与NKX同源框-1基因（NK2 homeobox 1, NKX2-1）和同源异形盒转录因子1（prospero homeobox protein 1, PROX1）形成复合体共同调控多个基因，涉及Notch信号通路、细胞周期等过程^[8]^。

## 2 Notch信号通路在SCLC中的作用

在肺神经内分泌肿瘤（pulmonary neuroendocrine tumor, PNET）的所有亚型中，SCLC的Notch信号通路的突变率显著高于其他亚型。Notch配体家族成员突变率较低（4%-7%），且这些突变是互斥的^[9]^。

### 2.1 Notch通路配体与受体的相互作用

来自临床试验人群的分析^[10]^证实，抑制性Notch配体DLL3在超过72.8%的SCLC中表达，并且大多数SCLC患者的DLL3表达水平较高。DLL3作为一种非典型的抑制性配体，其过表达可上调锌指蛋白转录因子Snail的表达，进而促进SCLC细胞的生长并增强其迁移和侵袭能力。DLL3还通过促进细胞增殖和获得对铂类双药化疗的耐药性，与PNET中转移性和治疗耐药表型有关^[11]^。其在SCLC肿瘤细胞上的广泛且特异性表达使其成为理想的治疗靶点。

Notch1通常表现为肿瘤抑制作用。Notch1激活导致G_1_期细胞周期生长停滞，显著降低肿瘤的增殖潜力^[12]^。Notch1还通过抑制上皮-间质转化（epithelial-mesenchymal transition, EMT）、促进细胞凋亡从而发挥多维度的抑癌作用^[13,14]^。Notch1的激活促进E-钙黏蛋白表达，增强细胞黏附，并抑制锌指蛋白转录因子Snail等EMT分子的表达，从而降低细胞的运动性和侵袭能力。相反，小干扰RNA（small interfering RNA, siRNA）敲低*Notch1*基因则可促进细胞侵袭^[13]^。Notch1表达的恢复可导致SCLC细胞出现上皮样区域，转染Notch1的SCLC细胞在小鼠模型中形成的肿瘤表现出上皮样分化及标志物减少，表明Notch1上调抑制了SCLC的NE分化^[14]^。

Notch1作为重要的肿瘤抑制因子，其功能失活在SCLC中发挥重要作用。临床分析^[9]^显示，约25%的SCLC病例存在Notch信号通路相关基因异常，其中29%的患者存在*Notch1*突变。在未检出基因突变的病例中，表观遗传调控机制可能发挥重要作用。表观遗传调控在经典SCLC细胞中通过组蛋白修饰抑制Notch1的表达，表现为该基因启动子区的组蛋白乙酰化水平降低。通过组蛋白去乙酰化酶抑制剂处理可恢复其表达，促进非NE表型转化^[15]^。

研究^[16]^发现，Notch2作为肺NE干细胞的标志性分子，在肺损伤修复中激活Notch信号通路下游靶基因*HES1*，驱动NE细胞去分化，并产生其他类型肺上皮细胞以促进上皮修复，表明Notch2可能与肿瘤起始活性与肿瘤内异质性（intratumor heterogeneity, ITH）有关。Notch3通过调控Jagged2配体的表达来调节Notch1，表明Notch1与Notch3信号通路可能在EMT的调控中存在相互作用。此外，实验表明Notch3可通过上调HES1蛋白表达水平抑制MDM2蛋白表达，进而上调Rb1和P21蛋白表达水平，抑制H446细胞增殖并阻滞H446细胞于G_0_/G_1_期^[17]^。

### 2.2 Notch信号通路的下游调控

*HES1*和*HEY1*是受NICD和免疫球蛋白kappa J区重组信号结合蛋白（recombination signal binding protein for immunoglobulin kappa J region gene, RBPJ）/MAML复合体调控的靶基因。*ASCL1*基因和*HES1*基因表达之间的平衡对于预测肺内肺部神经内分泌细胞（pulmonary neuroendocrine cell, PNEC）的谱系命运至关重要。研究^[18]^发现，*ASCL1*基因缺陷小鼠缺乏PNEC，而*HES1*基因缺陷小鼠的PNEC分化得到促进。这些发现表明，ASCL1与包括HES1在内的Notch信号通路协同诱导PNEC，从而影响PNEC细胞谱系。Notch通路活性降低时，可解除对ASCL1的抑制作用从而启动NE分化程序。ASCL1可促进SCLC的NE分化，而Notch信号通路则抑制NE分化，因此ASCL1和Notch1在SCLC中的表达常常是互斥的。

神经元限制性沉默因子（repressor element 1 silencing transcription factor, REST）为Notch信号通路的关键下游靶分子，介导了SCLC中NE向非NE的转化^[19]^。通过染色质免疫沉淀和功能实验发现，Notch1胞内结构域直接结合并激活REST的启动子，诱导其表达。REST通过转录抑制NE相关基因如泛素羧基末端水解酶L1（ubiquitin C-terminal hydrolase-L1, *UCHL1*）、血清降钙素基因相关肽（calcitonin gene-related peptide, *CGRP*）和*ASCL1*驱动细胞命运转变。*REST*基因的敲除显著削弱Notch介导的表型转换，这一过程具有不可逆性，可能与REST招募表观抑制复合物重塑染色质相关。此外，REST依赖的非NE细胞表现出化疗抗性，并通过分泌因子支持PNEC的生长，揭示了Notch信号通路在肿瘤异质性和肿瘤微环境（tumor microenvironment, TME）调控中的双重作用。靶向REST或可成为联合化疗抑制SCLC复发的潜在策略^[20]^。

### 2.3 多种信号通路参与Notch调控

基于转录因子ASCL1、NEUROD1、POU2F3和Yes相关蛋白1（Yes-associated protein 1, YAP1）的高表达，SCLC可分为四种亚型：SCLC-A、SCLC-N、SCLC-P和SCLC-Y。其中，SCLC-A是最常见的分子亚型，约占所有SCLC的70%^[21]^。在小鼠模型中*MYC*基因激活的Notch信号驱动SCLC从SCLC-A到SCLC-N最后到SCLC-Y的转化，仅Notch活性不足以驱动表型转换，*MYC*基因和Notch可能相互协作以驱动SCLC进展^[22]^。在*MYC*基因驱动的SCLC模型中，ASCL1的缺失显著抑制了肿瘤向SCLC-N的进展，并促使肿瘤细胞转变为SRY相关高泳动蛋白盒基因9（SRY-related high mobility group-box gene 9, *SOX9*）阳性的干样状态，具有类似神经嵴的特征^[23]^。类似于Notch，Hippo-YAP通路在SCLC中抑制NE分化和诱导耐药性。YAP通过上调Notch2促进REST的表达，从而促进NE向非NE转化^[19,24]^。YAP1的表达与Notch1、Notch2、Notch3及REST呈显著正相关，表明Hippo通路可能通过调控REST间接激活Notch信号。Notch配体DLL3的表达与YAP1呈显著负相关，提示Hippo通路可能通过抑制DLL3维持Notch信号的活性^[25]^。YAP、REST和Notch信号通路通过相互作用，共同调控SCLC中NE向非NE的转变^[19]^。

*ASCL1*基因是SCLC的细胞谱系特异性癌基因，在肺腺癌细胞系中诱导NE分化，并调节Wnt相关分子^[26]^。在SCLC细胞系中，ASCL1和Wnt11表达之间存在正相关，ASCL1通过在*Wnt11*基因增强子区域的赖氨酸H3K2乙酰化来调控Wnt11的表达。在SCLC细胞系中，Wnt11在ASCL1的调控下控制NE分化、细胞增殖和E-钙黏蛋白的表达，表明Notch信号通路可能与Wnt信号通路存在交互作用^[27]^。

纤维细胞通过分泌白细胞介素-6（interleukin 6, IL-6）激活Janus激酶2/信号转导和转录激活子3（Janus kinase 2/signal transducer and activator of transcription 3, JAK2/STAT3）信号通路，进而上调*c-MYC*基因的表达并激活Notch通路，驱动SCLC细胞从NE转向非NE^[28]^。视黄酸受体相关孤儿受体γ（retinoic acid receptor-related orphan receptor γ, RORγ）在SCLC中过表达，抑制RORγ活性显著降低了神经发生标志基因如*DLL3*的表达^[29]^。在帽蛋白抑制肌动蛋白动力学调节器（capping protein regulator of actin cytoskeleton dynamics, CRACD）缺失的SCLC模型中，Notch信号通路的活性显著下降，NE标志物上调^[30]^。这一过程可能与肌动蛋白骨架的紊乱从而抑制Notch信号通路的正常活性有关。综合上述，Notch信号通路与多种信号通路协同作用，推动SCLC从NE向非NE的转化，为SCLC治疗提供潜在靶点。

## 3 Notch信号通路在SCLC进展中的作用

### 3.1 Notch信号通路参与NSCLC至SCLC的转化

除了SCLC亚型之间的表型转换，Notch信号通路在非小细胞肺癌（non-small cell lung cancer, NSCLC）治疗后向SCLC的组织学转变中也起着关键作用。NSCLC在治疗后可能发生组织学转化为SCLC，这一过程涉及视网膜母细胞瘤基因（retinoblastomal, RB1）和肿瘤蛋白p53（tumor protein p53, TP53）的同时突变和Notch等信号通路的改变^[31]^。在肺腺癌向SCLC转化的过程中，Notch通路的基因（如*Notch1*、*Notch2*、*Notch3*）以及相关配体（如*JAG2*和*DLL4*）出现了下调，*ASCL1*表达上调，表明Notch信号的抑制可能促进了NSCLC向SCLC的转化^[32,33]^。在基底型鳞状细胞癌（basal squamous cell carcinoma, B-SqCC）中，Notch2的下调与*DLK1*、*ASCL1*等基因的上调共同作用，促进了肿瘤的NE特征。这些分子变化使B-SqCC在形态学和分子层面上表现出与SCLC更为显著的相似性^[34]^。因此，深入研究Notch信号通路及其相关分子的变化，可能为NSCLC转化为SCLC的机制提供新的见解，并为临床治疗策略的优化提供理论基础。

### 3.2 Notch通路与SCLC的ITH及耐药性

Notch信号通路的活性差异是驱动ITH的一个重要因素。Notch信号激活的SCLC细胞表现为生长缓慢和耐药非NE型，Notch信号抑制的SCLC细胞则维持NE特征^[20]^。ITH在肿瘤进展、转移及治疗耐药中发挥重要作用。NE亚型的肿瘤更易对复制应激靶向治疗产生反应，而非NE亚型主要组织相容性复合体I类分子（major histocompatibility complex I, *MHC-I*）基因表达较高，则更适合免疫治疗^[35,36]^。*CRACD*缺失下调*MHC-I*基因表达，诱导SCLC肿瘤细胞的可塑性和免疫逃逸^[30]^。值得注意的是，SCLC在出现化疗耐药性后重新表达MHC-I，提示免疫识别的恢复^[36]^。抑制赖氨酸特异性去甲基化酶1（lysine specific demethylase 1, LSD1）可激活Notch并抑制SCLC的NE特征，增加MHC-I表达，并增强对程序性细胞死亡受体1（programmed cell death 1, PD-1）抑制的反应^[37]^。

除了通过影响ITH来促进SCLC耐药性，Notch信号通路还直接参与调节SCLC对化疗的敏感度。近年来研究^[38]^发现，Notch信号通路的激活上调溶解性鸟苷酸环化酶的表达，进而激活cGMP/PKG信号通路，增加SCLC的耐药性。MYCNOS-SE是SCLC中一个重要的超增强子，MYCNOS-SE增强MYCNOS的转录，间接上调*MYCN*基因的表达，进而激活HES1的转录，增强Notch信号通路的活性，增强SCLC细胞的耐药性^[39]^。

### 3.3 Notch与SCLC相关TME Notch信号通路在SCLC

TME中发挥关键作用，特别是在肿瘤细胞间相互作用和协同效应方面。Notch激活的非NE肿瘤细胞可激活邻近NE肿瘤细胞中的Notch信号，并通过提供营养支持其生长^[20]^。非NE细胞通过旁分泌信号增强NE细胞的转移能力。NE细胞与非NE细胞共培养时，NE细胞表现出较强的增殖能力^[40]^。这种细胞间的协同作用在TME中形成双向促进效应，使NE和非NE细胞共同作用于肿瘤的侵袭性和转移性。

除了NE和非NE细胞，TME中其他细胞，如肿瘤相关成纤维细胞（cancer-associated fibroblasts, CAFs）、内皮细胞和免疫细胞等，也在肿瘤生长和转移中发挥重要作用。CAFs通过分泌IL-6，促进SCLC细胞向非NE转变。CAFs富集的SCLC样本中，观察到免疫细胞浸润增加，免疫激活标志物及PD-1、程序性细胞死亡配体1（programmed cell death ligand 1, PD-L1）的上调^[28]^。化疗通过调节Notch信号通路显著影响SCLC的TME。化疗后Notch信号抑制基因（如*DLL3*和*HES6*）在NE细胞中上调，可能在SCLC表型转变及TME重塑中发挥关键作用。化疗还促进了血管内皮生长因子（vascular endothelial growth factor, VEGF）信号通路在内皮细胞中的上调，增强血管生成，并促进成纤维细胞基质重塑及免疫细胞的抗肿瘤反应^[41]^。Notch信号通路在SCLC与免疫微环境的作用中呈现双重特征。一方面，*Notch1*基因突变与PD-L1表达呈负相关，从而减弱免疫逃逸机制^[9]^。另一方面，DLL3的高表达与PD-L1表达呈正相关，且常伴随中度至重度炎症浸润。PD-L1在DLL3表达阳性且无*Notch1*基因突变的患者表达更高，表明这类SCLC患者可能更易从免疫治疗中获益^[9,42]^。Notch信号通路在TME中的作用为理解肿瘤发展和治疗提供了新视角。

## 4 Notch信号通路的治疗前景

虽然Notch1、Notch2和Notch3在内的Notch受体被确定为SCLC中的肿瘤抑制因子，但发现抑制这些受体可以提高化疗的疗效。这可能与Notch受体的抑制阻断了Notch信号介导的NE到非NE的转换，并减少ITH和化疗耐药性有关。研究^[43]^发现，三氧化二砷（As_2_O_3_）可通过下调Notch1表达，诱导肺癌细胞的凋亡和细胞周期停滞并抑制肿瘤血管生成。这揭示了Notch信号的更多肿瘤抑制机制，并提供了新的治疗前景。此外，阻止*Notch*基因的表观遗传修饰也有助于SCLC的抑制。赖氨酸去甲基化酶5A（lysine demethylase 5A, KDM5A）作为表观遗传调控因子，通过抑制Notch2和Notch靶基因来维持ASCL1水平和NE分化^[44]^。组蛋白脱乙酰酶抑制剂丙戊酸被发现可介导Notch信号转导的激活并诱导SCLC细胞增殖停滞，表明对*Notch*基因表观遗传水平的调控值得进一步研究^[45]^。

DLL3作为Notch信号通路的抑制性配体，在约85%的SCLC细胞的表面显示异常表达，而在正常组织中表达极低^[46]^。其在SCLC肿瘤细胞上的广泛且特异性表达使其成为理想的治疗靶点。靶向DLL3的策略，包括抗体偶联药物（antibody-drug conjugates, ADC）、双特异性T细胞衔接器（bi-specific T cell engager, BiTE）和嵌合抗原受体T细胞免疫疗法（chimeric antigen receptor T-cell, CAR-T）等，在各期研究中取得了不同进展。Rova-T作为全球首个靶向DLL3的ADC药物，其I期研究数据显示总体患者客观缓解率为18%，但在后续两项关键III期试验MERU试验和TAHOE试验中均未体现临床优势^[47,48]^。此外，在TAHOE试验中，Rova-T治疗相关不良事件发生率升至约60%，显著高于I期观察到的38%基线水平，这直接导致该药物的研发终止。但一种新型的抗DLL3的ADC药物DB-1314在体内外细胞中展现出有效、持久和剂量依赖性的抗肿瘤作用，有望在临床环境中进行进一步评估^[49]^。

BiTE药物已在多种肿瘤适应证的临床开发中取得进展，在用于SCLC的治疗中，其中Tarlatamab最受瞩目。基于Tarlatamab的I期研究展现出良好的结局。II期研究对10 mg和100 mg这两种剂量水平的Tarlatamab治疗效果进行了比较分析。研究表明，两种剂量水平给药的客观缓解率和无进展生存期相当，但前者治疗相关不良事件的发生率更低^[50]^。该药物目前已进入III期临床阶段^[51]^。在三特异性T细胞衔接器（tri-specific T cell engager, TiTE）药物中HPN328单药治疗使39%受试者出现靶病灶缩小现象^[52]^。而靶向DLL3的CAR-T疗法AMG119在经治SCLC I期试验中，肿瘤缓解率达到20%，整体安全性可控^[53]^。这些药物都在靶向DLL3的治疗中展现出良好的治疗前景。

其他针对DLL3的其他非主流新疗法如[177Lu]Lu-DTPA-CHX-A"-SC16放疗也为SCLC的治疗提供了新的前景^[54]^。考虑到放疗对肿瘤免疫微环境的免疫调节作用，探索与ICIs的协同作用可能具有潜在益处。这一点在BiTE疗法上已获得证实，BiTE疗法与ICIs能够相互提高疗效，克服T细胞浸润不良和免疫治疗难治性肿瘤中的耐药性，从而形成良性循环^[55]^。化疗联合ICIs已被证明可提高SCLC生存率并改善疗效，但ICIs仅对一小部分SCLC患者有益^[56]^。因此，确定对ICIs治疗有持久反应的患者的预测性生物标志物并确定患者亚群对免疫治疗的敏感性是十分重要的。研究^[57]^证实，YAP-1高表达的SCLC-Y亚型对免疫治疗的获益最大，可能与YAP1上调PD-L1表达，募集M2巨噬细胞、髓源性抑制细胞、调节性T细胞，抑制自然杀伤细胞等途径营造免疫抑制环境有关。此外，其他分子亚型也有其潜在治疗靶点。SCLC-A亚型以DLL3和B细胞淋巴瘤因子2（B-cell lymphoma 2, BCL2）为核心靶点，目前BCL2抑制剂拟作为靶向SCLC-A的药物正在研究中^[58]^；SCLC-N亚型依赖MYC，高MYC水平会提高SCLC肿瘤细胞对极光激酶A（aurora kinase A, AURKA）和极光激酶B（aurora kinase B, AURKB）抑制剂的敏感性，从而使肿瘤细胞增殖受到抑制^[59]^；SCLC-P亚型对靶向作用于DNA损伤反应通路的聚腺苷二磷酸核糖聚合酶抑制剂（poly ADP-ribose polymerase inhibitors, PARPi）敏感，其敏感性可被DNA损伤修复调节因子Schlafen家族成员11（Schlafen family member 11, SLFN11）预测^[58-60]^。

## 5 总结与展望

本文综述了Notch信号通路在SCLC中的作用，重点探讨了其在SCLC表型转换、耐药性和TME中的关键作用。Notch信号的失调与SCLC的发生和发展密切相关，既影响肿瘤的增殖和分化，又在肿瘤免疫逃逸和ITH中发挥重要作用，为未来的治疗提供了新的靶点。

未来的研究应深入探讨Notch信号通路在SCLC中的复杂调控机制，尤其是与Hippo-YAP等其他信号通路的交互作用。针对Notch通路的靶向药物研发将是未来研究的重点，特别是在克服现有治疗耐药性及提高临床疗效方面。随着分子标志物和基因组学研究的进展，精准医疗与个性化治疗有望进一步提高SCLC治疗效果，为临床治疗提供更有效的策略。
